# NK Cells as Potential Targets for Immunotherapy in Endometriosis

**DOI:** 10.3390/jcm8091468

**Published:** 2019-09-14

**Authors:** Aneta Ścieżyńska, Michał Komorowski, Marta Soszyńska, Jacek Malejczyk

**Affiliations:** 1 Department of Histology and Embryology, Medical University of Warsaw, Chałubińskiego 5, 02-004 Warsaw, Poland; asciezynska@wum.edu.pl; 2 Laboratory for Experimental Immunology, Military Institute of Hygiene and Epidemiology, 01-163 Warsaw, Poland; michalpiotrkomorowski91@gmail.com (M.K.); marta.soszynska@onet.eu (M.S.)

**Keywords:** endometriotic cells, endometriosis, immune checkpoint, immunotherapy, major histocompatibility complex class I, natural killer (NK) cells, NK cell receptors

## Abstract

Endometriosis is a common gynecological disease defined by the presence of endometrial-like tissue outside the uterus, most frequently on the pelvic viscera and ovaries, which is associated with pelvic pains and infertility. It is an inflammatory disorder with some features of autoimmunity. It is accepted that ectopic endometriotic tissue originates from endometrial cells exfoliated during menstruation and disseminating into the peritoneum by retrograde menstrual blood flow. It is assumed that the survival of endometriotic cells in the peritoneal cavity may be partially due to their abrogated elimination by natural killer (NK) cells. The decrease of NK cell cytotoxic activity in endometriosis is associated with an increased expression of some inhibitory NK cell receptors. It may be also related to the expression of human leukocyte antigen G (HLA-G), a ligand for inhibitory leukocyte immunoglobulin-like receptor subfamily B member 1 (LILRB1) receptors. The downregulated cytotoxic activity of NK cells may be due to inhibitory cytokines present in the peritoneal milieu of patients with endometriosis. The role of NK cell receptors and their ligands in endometriosis is also confirmed by genetic association studies. Thus, endometriosis may be a subject of immunotherapy by blocking NK cell negative control checkpoints including inhibitory NK cell receptors. Immunotherapies with genetically modified NK cells also cannot be excluded.

## 1. Introduction

Endometriosis is a common estrogen-dependent benign gynecological disorder affecting approximately 10% of women in their reproductive age which corresponds to 200 million women worldwide [1,2]. The disease is defined by the presence and growth of endometrial-like tissue (endometrial glands and stroma) in ectopic sites most frequently on the pelvic viscera (peritoneal endometriosis), ovaries (ovarian endometriosis) or in rectovaginal septum (deep infiltrating endometriosis). This ectopic endometrial-like tissue is commonly referred to as endometriotic tissue. Endometriotic tissue is typically composed of endometrial stromal cells, endometrial epithelial cells and displays signs of bleeding manifested by numerous erythrocytes and hemosiderin-positive macrophages. The foci of endometriotic tissues are usually surrounded by fibrotic tissues [3,4].

Endometriosis is associated with pelvic inflammation and is most commonly manifested by dysmenorrhea, chronic pelvic pains, dyspaurenia, bowel upset, dyschezia and dysuria as well as subfertility and infertility [5,6,7]. The current treatments are limited to analgesic or hormonal therapy and surgery [1,2,8]. Endometriosis is a debilitating disease that significantly affects the quality of life and thus constitutes an important social and clinical problem.

Endometriosis is a complex disease that depends on a variety of still poorly defined genetic, immune and environmental factors [1,2,9,10]. The origin and the mechanisms of the development of ectopic endometriotic lesions still remains a matter of dispute [3,9,10,11,12]. The most accepted Sampson’s theory (implantation theory) claims that foci of ectopic endometriotic tissue originates from endometrial cells exfoliated during menstruation and disseminated into the peritoneum by retrograde tubal flow [13]. However, it cannot be excluded that endometriosis may also develop as a result of coelomic metaplasia of Müllerian system remnants [14,15].

There is a growing bulk of evidence that endometriosis is related to the deviations of the local and systemic immune system. The disease manifests by abrogated cellular and humoral immune responses including peritoneal infiltration with immune cells, the activation of macrophages, abnormal lymphocyte responses and abrogated natural killer (NK) cell cytotoxicity as well as excessive production of proinflammatory and regulatory cytokines [16,17,18]. Endometriosis is also associated with an elevated production of a variety of autoantibodies such as anti-nuclear, anti-phospholipid, and anti-endometrial antibodies [18,19,20,21]. It may be argued for endometriosis to be an autoimmune/autoinflammatory disorder. However, the role of autoimmunity in the development of endometriosis still remains a matter of dispute.

Retrograde menstruation which is considered as a major way of dissemination of endometriotic cells occurs in almost all women but only a proportion of them (ca. 10%) develop endometriosis. Thus, the question arises what may facilitate endometriotic cell survival, implantation and growth in the ectopic environment. It has been demonstrated that the cells from endometriotic lesions are more resistant to apoptotic cell death [22,23,24]. They also display an increased adhesiveness and invasiveness that may account for a higher rate of invasion and implantation [22,25,26,27]. The development of ectopic endometriotic lesions may involve the participation of endometrial stem/progenitor cells [28] as well as may be facilitated by epithelial-to-mesenchymal transition [29]. Furthermore, the development and progression of endometriotic lesions may be facilitated by the induction of local angiogenesis [16]. Thus, endometriotic lesions share some typical features with tumor cells.

It is possible that the survival and growth of endometriotic cells in the peritoneal cavity is also due to their abrogated recognition and elimination by local immune cells, such as macrophages and NK cells. Indeed, the evidence accumulates that endometriosis may be associated with downregulated NK cell cytotoxicity [19,30,31,32]. The mechanisms of this abrogation are still poorly understood. The question, to what extent abrogation of NK cells may contribute to development of endometriosis, also remains unanswered.

**Figure 1:**
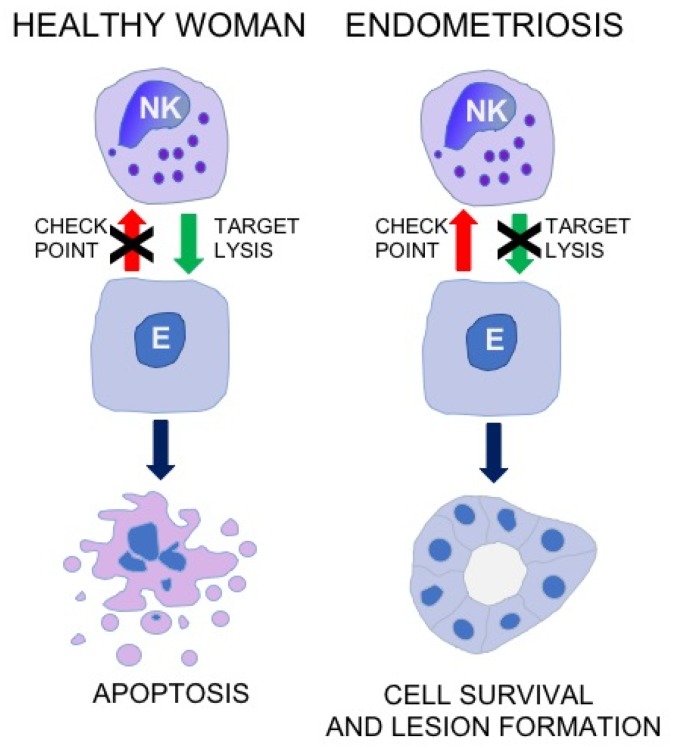
The concept for the role of NK cell immune checkpoints in the immunopathogenesis of endometriosis. NK, natural killer cells: E, endometriotic cell.

According to the concepts of regulation of immune responses, the activity of NK cells may depend on the engagement of a battery of immune checkpoint molecules [33,34,35]. It is widely accepted that checkpoint molecules may play a pivotal role e.g., in the escape of neoplastic cells from under control of the immune system. Accordingly, abrogated elimination of shed endometrial cells in the course of endometriosis may be associated with the activation of some checkpoint molecule pathways (Figure 1).

The identification of checkpoints responsible for downregulated NK cell activity in the course of endometriosis may be useful for the identification of new possible therapeutic targets. Therefore, the aim of the present review was to summarize the knowledge on the status of NK cell cytotoxicity in patients with endometriosis and the putative mechanisms of endometriotic cell escape from under NK cell surveillance, as well as to reveal the possibilities for targeted NK cell immunotherapy of the disease.

## 2. Phenotype and Function of NK Cells

NK cells are defined as large granular lymphocytes displaying cluster of differentiation (CD)3^-^ CD56^+^ CD16^+/-^ CD57^+/-^ phenotype. They are able to spontaneously recognize and kill a variety of virus-infected, neoplastic and stressed cells, thus being responsible for innate surveillance against viral infections and cancer [36,37,38]. NK cells may also recognize some normal cell types as well as play a part in the regulation of other phenomena, including antigen presentation, autoimmunity, inflammation, transplant rejection, and pregnancy [39,40,41,42,43,44].

NK cells comprise two major subpopulations depending on expression of CD56 and CD16 markers [45,46,47]. CD56^dim^ CD16^+^ cells displaying high cytotoxic potential account for approximately 90% of all circulating NK cells. On the other hand, CD56^bright^ CD16^-^ subset plays a regulatory role producing high amounts of cytokines including IFN-γ and TNF. It is believed that CD56^bright^ cells represent less differentiated NK cells that upon the stimulation may acquire mature CD56^dim^ CD16^+^ phenotype and become highly cytotoxic [47]. Both populations also differ in a spectrum of other markers including a variety of NK cell receptors. The phenotype and function of NK cells may also be determined by their tissue location [38,46]. In the context of the present paper, of special interest may be CD56^bright^ uterine NK (uNK) cells which are a major lymphocyte population in normal endometrium [46,48,49]. These cells locally develop from CD34^+^ precursors and seem to participate in spiral arteries modelling, placenta development and pregnancy maintenance.

The cytotoxicity mechanisms involve the induction of apoptosis in target cells by the release of content of cytolytic granules [50] or by FasL-mediated mechanism via triggering of apoptotic Fas (CD95) receptor [51]. Natural cytotoxicity is also an attribute of natural killer T cells (NKT cells) which share many similarities with classical NK cells [52].

The recognition of target cells by NK cells involves the participation of some adhesion molecules [53,54] as well as a battery of different stimulatory and inhibitory receptors belonging to several different receptor families, such as killer immunoglobulin-like receptors (KIRs) [55,56,57], leucocyte immunoglobulin-like receptors (LILRs) [58,59], natural cytotoxicity receptors (NCRs) [60,61], and killer cell lectin-like receptors (KLRs) comprising CD94/natural killer G2 (NKG2) [62,63] and natural killer receptor P1 (NKRP1) subfamilies [64,65,66]. It appears that triggering of a NK cell cytotoxic reaction is determined by a balance between the activation and inhibition signals [67]. The major NK cell inhibitory receptors which may be considered as potential NK cell immune checkpoints are shown in Table 1.

## 3. Cytotoxic Activity of NK Cell in Patients with Endometriosis

A decreased NK cell activity in patients with endometriosis was reported for the first time by Oosterlynck et al. who found lowered cytotoxicity of NK cells against K562 cells both in the peripheral blood and the peritoneal fluid [68]. The decreased lysis of K562 cells by peripheral blood NK cell from women with endometriosis was further confirmed by other investigators [69,70,71,72,73,74]. The abrogated lysis of K562 was also reported in the case of peritoneal NK cells [75,76,77]. Nevertheless, it should be stressed, that in some studies abrogated cytotoxicity of peripheral blood or peritoneal fluid NK cells could not be confirmed [76,78,79,80]. Some of these negative results, however, may be explained by the low number of patients and controls.

The majority of studies on NK cell cytotoxic activity in endometriosis has been performed on K562 human leukemia cells serving as a universal target [81]. It should be stressed, however, that K562 cells are lacking the expression of major histocompatibility (MHC) class I molecules [82] that renders them much more sensitive to NK cells than normal cells endowed with MHC class I molecules. Therefore, in the case of endometriosis, it seems more reasonable to perform cytotoxicity assays using endometrial or endometriotic epithelial or stromal cells as targets. Unfortunately, endometrial cells were used as targets only in two independent studies [69,80]. Both studies showed that NK cell activity against endometrial cells was decreased in women with endometriosis as compared to the control subjects. These results, however, need further confirmation.

## 4. Frequency of NK Cell in Patients with Endometriosis

The abrogated NK cell activity might be partially due to the disturbance of numbers of circulating effector cells. However, in the majority of studies, the evaluation of peripheral blood NK cells in patients with endometriosis has not revealed any significant change in the frequency of CD56^+^ and/or CD16^+^ NK cells when compared to healthy control women [69,70,74,75,79,80,83,84,85,86,87,88]. Only two studies showed a decreased frequency of CD57^+^CD16^+^ or CD56^+^/CD16^+^ NK cells [89,90]. It should be mentioned that there were also two publications reporting increased frequency of CD56^+^ or CD56^+^/CD16^+^ NK cells [91,92]. Similar results were obtained with peritoneal NK cells. Generally, no change of percentage of CD56^+^ and/or CD16^+^ cells was observed in peritoneal fluid of patients with endometriosis [75,79,84,86,87,88,92]. Nevertheless, one paper reported an increase of CD56^+^/CD16^+^ [90] and two papers reported decreased levels of peritoneal CD56^+^ NK cells [93,94].

There is only a limited information on NK cells infiltrating the eutopic endometrium and ectopic endometriotic tissue of patients with endometriosis. Klentzeris et al. [95] reported no differences in the numbers of CD16^+^ uNK cells present in the eutopic endometrium in patients with endometriosis as compared to normal women. Giuliani et al. [96] showed that the numbers of CD16^+^ cells and NKp46+/CD56+ cell ratio were increased in eutopic endometrium of women with unexplained recurrent spontaneous abortion and infertility as compared to healthy fertile women. These differences, however, were unrelated to endometriosis. Furthermore, there were no changes in CD56^+^ cells. On the other hand, there are reports showing that the numbers of CD56^+^ or CD16^+^ cells in endometriotic tissues are lower than in eutopic endometrium from healthy women [90,97] and that NK cells from endometriotic lesions do not show typical changes characteristic to uNK cells in the eutopic endometrium [98].

The above cited results imply that abrogated NK cell activity in patients with endometriosis is not due to the diminished numbers of circulating effector cells. It is possible, however, that impaired elimination of endometriotic cells may be related to their lower infiltration of endometriotic tissue.

## 5. Expression of NK Cell Receptors and Their Ligands in Patients with Endometriosis

The impaired NK cell cytotoxicity in patients with endometriosis may be due to the abrogated expression of stimulating and inhibiting NK cell receptors. Several independent studies exist showing that endometriosis is associated with the increased expression of some inhibitory NK cell receptors. The best evidenced is the increased expression of human leukocyte antigen (HLA)-C2 recognizing inhibitory KIR2DL1 receptor by peripheral blood and/or peritoneal NK cells [76,79,84,86,99,100]. On the other hand, there was no upregulation of the expression of KIR2DL2/3 bound by HLA-C1 ligands [76,84,86,99]. The expression of KIR3DL1 on peripheral blood was unchanged whereas its expression on peritoneal NK cell was reported to be increased [76].

There was also no change in the expression of KIR2DL4 receptor recognizing HLA-G ligands [100]. Unlike other long-tailed KIRs, KIR2DL4 has only one immunoreceptor tyrosine-based inhibitory motive (ITIM) and is endowed with positively charged arginine residue in the transmembrane domain that may recruit adaptor proteins with immunoreceptor tyrosine-based activating motives (ITAM) [56,101]. Thus, KIR2DL4 appears to be rather a weak stimulatory receptor with a predominant ability to trigger cytokine production by NK cells [101]. KIR2DL4/HLA-G interaction seem to play a part during pregnancy, however, its biological and clinical significance still remains a matter of controversies [101]. Its role in endometriosis also remains unknown.

The KIR family of receptors recognizes MHC class I molecules. Thus, their engagement in cytotoxicity reactions against endometriotic cells depends strictly on their presence or absence on the surface of the target cells. The MHC class I molecules are constitutively expressed in all nucleated cells thus they also serve as constitutive ligands on endometriotic cells. Interestingly, the expression of MHC class I molecules was reported to be upregulated in eutopic endometrium of women with endometriosis as compared to healthy controls [102]. The evaluation of soluble HLA class I molecules in sera of endometriosis patients has revealed no significant differences when compared to healthy women [103]. However, Matalliotakis et al. have reported that the levels of circulating soluble HLA class I molecules are decreased in endometriosis patients [104]. The significance of these findings awaits elucidation.

Of special interest appears to be the specific expression of HLA-G molecules which are ligands for KIR2DL4 and inhibitory LILRB1 receptors. HLA-G are non-classical MHC class I molecules that are almost exclusively expressed in placenta where they seem to protect fetal tissues from maternal immune cells [105,106]. They may be also found in circulation as soluble molecules (sHLA-G) where they have been found to be associated with better pregnancy rate [107]. Although HLA-G has been initially reported to be absent in endometria and endometriotic tissue [108], many other studies showed that HLA-G is expressed by endometrial and/or endometriotic cells, preferably by epithelial gland cells [109,110,111]. It has been also found that expression of HLA-G in eutopic endometrium may be restricted to menstrual period [100].

The sHLA-G molecules were found to be present both in serum and peritoneal fluid of control women and patients with endometriosis [111,112]. In comparison to healthy control, the sHLA-G levels were increased in serum but not the peritoneal fluid of women with advanced stage of endometriosis [111]. No increase of peritoneal sHLA-G was also observed in other previous study [112]. This may imply that sHLA-G molecules do not play a significant role in the pathogenesis of endometriosis. It should be stressed, however, that the functional role of HLA-G in the context of KIR2DL4 and LILRB1 receptors in course of endometriosis remains unknown and requires further investigations.

The analysis of NK cells from peripheral blood and peritoneal fluid from women with endometriosis did not reveal any change of expression of CD94 (KLRD1) [99]. This forms heterodimeric receptors with other members of the CD94/NKG2 family, i.e., NKG2A and NKG2C chains [64]. There was no change in the expression of the inhibitory NKG2A receptor by peripheral blood NK cells, however its expression on NK cells from the peritoneal fluid was increased in patients with endometriosis [88]. The same authors did not find any change of expression of the stimulatory NKG2C receptor [88]. Both NKG2A and C receptors recognize MHC class Ib HLA-E molecules [64,113]. However, there is no published information on HLA-E expression by the endometriotic cells. The HLA-E molecules assembly with peptides derived from the signal sequences of other class I MHC molecules, thus the HLA-E expression may depend on the rate of other MHC class I expression [113]. The increased expression of inhibitory NKG2A receptor may constitute one of the NK cell checkpoints and may contribute to the immunopathogenesis of endometriosis, therefore, further investigations on the HLA-E expression in endometriotic tissues are strongly encouraged.

Endometriosis was also reported to be associated with a decreased expression of stimulatory NKG2D molecule [114] recognizing the non-classical MHC class I ligands belonging to the MIC and ULBP family [62,115]. However, only one published study exists on the evaluation of soluble MICA, MICB and ULBP-2 in the peritoneal fluid of patients with endometriosis [116]. In patients who displayed detectable amounts of these molecules, the levels of MICA and MICB were significantly upregulated as compared to the control subjects and correlated with the disease severity. However, there were no similar differences in the levels of ULBP-2 [116]. This may suggest that soluble MICA and MICB may downregulate NK cell activity against endometriotic cells. Nevertheless, this assumption needs further verification in replicate studies.

The inhibitory NKRP1A receptor belonging to KLR family and being activated by lectin-like transcript 1 (LLT1) ligand appears to be another important NK cell checkpoint [65,66]. However, the authors were unable to find any information on a putative role of NKRP1A/LLT1 checkpoint in the pathogenesis of endometriosis. This system may be of special interest because it has been found that the expression of LLT1 protects many different tumor cells from lysis by NK cells and the downregulation of this molecule resulted in the restoration of target cell killing [66]. A similar effect was also observed following the downregulation of NKRP1A expression in NK cells, e.g., by their stimulation with IL-2 [117]. Thus, the studies on the possible role of NKRP1A and LLT1 in endometriosis may be of great interest.

Cancer studies have revealed that another important checkpoint molecule involved in the escape of tumor cells from under immune surveillance is programmed death protein 1 (PD-1) receptor and its programmed death ligand 1 (PDL-1) [33,34,55]. However, the information of the possible role of PD-1/PDL-1 interactions in the course of endometriosis is very limited. Recently, it has been shown that the expression of both PD-1 and PDL-1 is upregulated in CD4^+^ and CD8^+^ T cells and CD19^+^ B cells from the peripheral blood of patients with endometriosis as compared to control women [118]. The increased expression of PD-1/PDL-1 in CD4^+^CD8^+^ T cells was confirmed by Wu et al. [119]. More importantly, the latter authors showed that PD-1/PDL-1 is also expressed in the eutopic endometrium and ectopic endometriotic lesions and that this expression is increased in endometriosis. Furthermore, they found that PDL-1 in eutopic endometrial cells may be upregulated by 17-β-estradiol. The significance of this finding in the context of impaired NK cell activity needs further investigations.

It should be stressed that the target cell recognition by the NK cell and formation of cytolytic synapse requires the participation of adhesion molecules, especially the interaction between leukocyte function antigen 1 (LFA-1) and intercellular adhesion molecule 1 (ICAM-1) molecules [53,54]. Interestingly, soluble ICAM-1 (sICAM-1) molecules may act as competitors and block LFA-1-dependent NK cell target recognition. Accordingly, it has been reported that cultured endometrial cells may shed sICAM-1 molecules which in turn inhibit NK cell cytotoxicity [120,121]. Furthermore, it was found that cultured endometrial cells from patients with endometriosis release more sICAM-1 than normal endometrial cells, however the levels of sICAM-1 were not increased in the peritoneal fluid of endometriosis patients [121]. On the other hand, Fukaya et al. showed that levels of sICAM-1 in the peritoneal fluid of endometriosis patients were increased and interfered with NK cell cytotoxicity [122]. The serum levels of sICAM-1 in patients with endometriosis were reported to be decreased [84] or unchanged [103]. Thus, it appears that sICAM-1 may be considered as yet another factor that may affect the recognition and killing of endometriotic cells.

## 6. The Role of Local Factors in Regulation of NK Cell Activity in Endometriosis

The mechanisms responsible for the regulation of the expression of NK cell receptors and their ligands in course of endometriosis still remain obscure. Peritoneal milieu and endometriotic tissues in women with endometriosis may contain a bulk of inhibitory cytokines that may interfere with normal immune responses [123]. It has been reported that NK cell cytotoxicity may be inhibited by the peritoneal fluid [94,114,124,125] and serum [126] from endometriosis patients as well as supernatants from the culture of endometriotic tissue explants [127]. It is plausible that one of the multiple factors that may be involved in the inhibition of NK cell cytotoxicity in the course of endometriosis is transforming growth factor β (TGF-β). This cytokine exerts pleiotropic effects on many cells and tissues and the evidence grows that it plays a pivotal role in the pathogenesis in endometriosis [128]. It has been reported that peritoneal platelet-derived TGF-β exerted multiple inhibitory effects upon NK cells, including the suppression of their cytotoxic activity and the inhibition of stimulatory NKG2D receptor expression [114].

Furthermore, the downregulation of NK cell cytotoxicity by peritoneal fluid from women with endometriosis was also found to be attributed to interleukin 6 (IL-6) which was responsible for the inhibition of NK cell cytotoxicity and the downregulation of granzyme B and perforin expression [94]. Other cytokines which may be involved in the downregulation of NK cell function in endometriosis is endometriotic tissue-derived IL-15 [129,130]. This cytokine was reported to downregulate granzyme B and interferon production by NK cells as well as decreased the expression of stimulatory NKG2D and NKp44 NK cell receptors [130]. This inhibitory role of IL-6 and IL-15 remains to be further elucidated inasmuch as both IL-6 [131,132] and IL-15 [133,134] were also reported as stimulators of cytotoxic activity and expansion of NK cells.

It was also reported that peritoneal fluid from patients with endometriosis contains increased concentrations of p40 subunit of IL-12 that may counteract a stimulatory effect of heterodimeric IL-12 on NK cell activation [135]. Endometriosis is also associated with increased levels of inhibitory IL-10 [123]. This cytokine has been reported to be a potent inhibitor of NK cells [136], thus its role in the downregulation of NK cell cytotoxicity in endometriosis cannot be excluded. These results strongly imply that the inhibition of NK cell cytotoxicity in the course of endometriosis may be in part related to factors released by the peritoneal and endometriotic cells. Although TGF-β appears to be the most possible factor engaged in the abrogation of local and systemic NK cell activity in endometriosis, the role of other, yet unidentified factors cannot be excluded.

## 7. Association of Endometriosis with Haplotypes and Polymorphisms in NK Cell Receptor Genes

The triggering of NK cell cytotoxicity requires a combination of stimulatory and inhibitory receptors and their specific ligands expressed on the target cells. In particular, this is important in the case of polygenic KIR and LILR complex and their respective HLA ligands where it may determine susceptibility to some diseases [59,137].

KIR complex comprises of 14 genes and is characterized by the very high diversity related to the individual combination of specific KIR genes and their polymorphism. There exist over 30 haplotypes differing in the number and type of KIR genes which are classified into two distinct groups. Group A haplotype is composed of a fixed cluster of 9 genes in which inhibitory KIRs dominate. On the contrary, group B haplotypes are highly variable in both the number and combinations of KIR genes. It appears that the stimulation or inhibition of NK cell activity is attributed to a given haplotype combination, where the KIR AA haplotype appears to be the most inhibitory [56,57,138].

There are no studies showing the association of endometriosis with a particular KIR haplotype. However, Kitawaki et al. have found that endometriosis in Japanese women is associated with a decreased frequency of *KIR3DS1* gene coding for a stimulatory receptor [139]. Furthermore, the analysis of the combinations of KIR genes with their corresponding HLA genes showed that endometriosis is also associated with an increased frequency of inhibitory KIR/HLA class I gene combinations. In the Polish population, endometriosis was reported to be associated with a lower frequency of *KIR2DS5* gene coding for an inhibitory receptor, thus suggesting a protective role of this gene [140]. A further study has revealed that there is no direct association with other KIR complex genes and a protective role of *KIR2DS5* can be seen only in women carrying HLA-C2 group genes, in particular, those with peritoneal localization of the disease [141]. Interestingly, a lowered risk of peritoneal localization and a minimal/mild stage of the disease may be also associated with *KIR2DS5/KIR2DS4del* genotype. Both genes are in a strong negative linkage disequilibrium, therefore, the association with *KIR2DS4del* may be explained by an indirect role of the absence of *KIR2DS5* gene [141].

The analysis of the polymorphism of inhibitory *LILRB1* and *LILRB2* genes showed that endometriosis in the Polish population is associated with an increased frequency of 5651AA (5651G > A; rs41308748) genotype of *LILRB1* gene [142]. The advanced (moderate/severe) stages of the disease were also associated with 59AG genotype (59A > G; rs383369) of *LILRB2* gene [142].

The receptors of the KIR and LILR complex are bound and triggered by specific MHC class I molecules. Therefore, endometriosis might be also associated with some HLA class I genes. Kiwataki et al. [143] found that the frequency of HLA-Cw*0702 belonging to the HLA-C1 group serving as ligands for KIR2DL2/3 and KIR2DS4 receptors increased in Japanese patients with endometriosis as compared to healthy control women. However, no association with any of the HLA-A, -B or -C genes has been reported by other investigators [141,144,145]

The analysis of polymorphisms of *HLAG* gene coding for a ligand for KIR2DL4 and LILRB2 receptors has revealed that endometriosis in Polish women is associated with a lower frequency of its −964GG genotype (−964A > G; rs1632947) [142]. A further analysis has shown that a decreased frequency of −964GG as well as −725CT (−725C > G > T; rs1233334) *HLAG* genotype is associated with a minimal/mild stage of the disease, and −964GG genotype is associated with peritoneal endometriosis. These protecting polymorphisms may be associated with a decreased expression of *HLAG* gene, thus arguing for the role of HLA-G molecule in the abrogated mechanism of NK cell cytotoxicity. It should be stressed however, that endometriosis was found not to be not associated with the polymorphism of KIR2DL4 gene which also serves as an inhibitory receptor for HLA-G [142].

In conclusion, endometriosis may be associated with a decreased frequency of *KIR3DS1* and *KIR2DS5* genes coding for the activating receptors which suggests that the expression of these receptors may protect from the disease. On the other hand, susceptibility to the disease may be related to an allelic variant of *LILRB1* coding for the inhibitory receptor for HLA-G. Endometriosis is also associated with allelic variants of *HLAG,* thus suggesting that the LILRB1/HLA-G interaction may play a part in the inhibition of NK cell activity and the development of the disease.

## 8. Conclusions and Prospects for Immunotherapy

**Figure 2:**
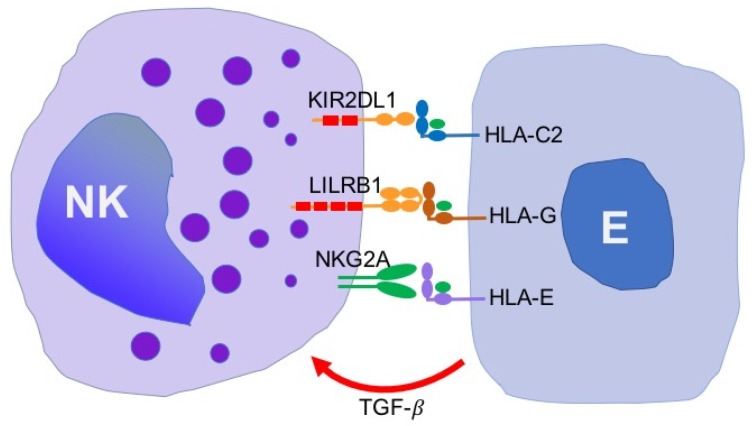
NK cell receptors and ligands identified so far, as possible check points responsible for the inhibition of lysis of endometriotic cells. NK, natural killer cells; E, endometriotic cell; KIR, killer immunoglobulin-like receptor; HLA, human leukocyte antigen; NKG, natural killer G2; TGF-β, transforming growth factor β.

The present review shows evidence that endometriosis is associated with the downregulation of NK cell cytotoxic activity that may account for the abrogated elimination of the disseminating endometrial/endometriotic cell. Functional and genetic studies have revealed that possible mechanisms underlie the lysis of endometriotic target cells which include the participation of KIR, LILRB and NKG2 family of receptors recognizing MHC class I molecules. The specific inhibitory NK cell receptor/ligand interactions that have been identified so far, as possible checkpoints for the elimination of endometriotic cells are shown on Figure 2.

These checkpoints include KIR2DL1 receptor which recognizes HLA-C2 group of MHC class I molecules as well as LILRB1 receptors specific for HLA-G. The latter interaction may be of special interest since HLA-G appears to be specifically expressed on endometrial and endometriotic cells. To the authors’ knowledge, no specific therapies disrupting LILRB1 interactions with HLA-G have been reported so far. This possibility, however, should deserve some special attention.

It is also possible that endometriosis is associated with the upregulated expression of the inhibitory NKG2A receptor that is bound by HLA-E molecules. HLA-E molecules are expressed by many tumor cells, however, the expression of HLA-E molecules on endometriotic cells has not been confirmed as yet. Nevertheless, the use of anti-NKG2A antibodies in the treatment of some tumor types has been reported to exert promising therapeutic effects [35]. Thus, the use of anti-NKG2A antibodies might be also effective in the immunotherapy of endometriosis.

Of great interest may be also the NK cell checkpoint involving the PD-1/PDL-1 interaction. Recently, PD-1 and PDL-1 were reported to be expressed on the cells from the endometriotic lesions and both molecules were found to be upregulated on the circulating lymphocytes from women with endometriosis. This information requires confirmation, nevertheless, the disruption of the PD-1/PDL-1 interaction appears to be a very promising target for immunotherapy of endometriosis. This possibility is strongly supported by encouraging results of cancer immunotherapy with specific anti-PD-1 and ant-PDL-1 antibodies [33,146,147].

The upregulation of inhibitory NK cell receptors and the downregulation of stimulatory ones in the course of endometriosis may be related to the activity of some local inhibitory factors, e.g., TGF-β. Thus, it is tempting to speculate whether the inhibition of NK cell cytotoxicity might be reversed by anti-inhibitory cytokine therapy. Alternatively, the inhibition of NK cells might be overcome by some NK cell stimulatory cytokines, e.g., IL-2. The stimulation of NK cells with IL-2 generates the so called lymphokine activated killer (LAK) cells which display increased cytotoxic potential against a variety of resistant tumour cells, and therefore, may be used in adoptive therapy of some types of cancer [148]. Interestingly, LAK cells were found to exert an increased cytotoxicity against various targets including endometrial cells in women with endometriosis [74]. It has been also reported that IL-2 resulted in the activation of host immune reactivity and further resulted in the reduction of endometrial implants in an experimental rat model of endometriosis [149]. Thus, stimulatory cytokine therapy appears to be another option for the treatment of endometriosis.

It should be also mentioned that the most current cancer immunotherapies include the application of genetically modified NK (CAR-NK) cells carrying chimeric antigen receptors that specifically target tumor cells [150,151,152]. These CAR-NK cells are presently tested in various preclinical and clinical studies and it is tempting to speculate that they might also serve as an effective tool in immunotherapy of endometriosis.

By designing NK cell-based immunotherapy of endometriosis, it must not be forgotten that the activity of these cells depends on a delicate balance between the activating and inhibiting receptors which may constitute a serious risk of unwanted side effects. This may include e.g., systemic overactivation of NK cells possibly leading to tissue injuries. Accordingly, anti-PD-1/anti-PDL-1 therapy was reported to induce a plethora of adverse effects affecting a variety of tissues and organs [153,154]. Thus, the selection and application of specific immunotherapy in endometriosis must be done with caution.

## Figures and Tables

**Table 1:** Major NK cell inhibitory receptors that may act as immune checkpoint molecules and their ligands.

Receptor Family	Inhibitory Natural Killer (NK) Cell Receptors	Ligands
Killer immunoglobulin-like receptors (KIR)	KIR2DL1	Human leukocyte antigen (HLA)-C2 group
	KIR2DL2/3	HLA-C1 group
	KIR3DL1	HLA-Bw4 serotypes
	KIR3DL2	HLA-A3/11 serotype
Leucocyte immunoglobulin-like receptors (LILR)	LILRB1	HLA-A, -B, -C, -G
Cluster of differentiation (CD) 94/Natural killer G2 (NKG2)	NKG2A	HLA-E
Natural killer receptor P1 (NKRP1)	NKRP1A	Lectin-like transcript 1 (LLT1)
	Programmed death 1 (PD-1)	Programmed death ligand 1 (PDL-1)
